# Gastrointestinal Dynamics of Non-Encapsulated and Microencapsulated *Salmonella* Bacteriophages in Broiler Production

**DOI:** 10.3390/ani12020144

**Published:** 2022-01-08

**Authors:** Laura Lorenzo-Rebenaque, Danish J. Malik, Pablo Catalá-Gregori, Clara Marin, Sandra Sevilla-Navarro

**Affiliations:** 1 Departamento de Producción y Sanidad Animal, Salud Pública Veterinaria y Ciencia y Tecnología de los Alimentos, Instituto de Ciencias Biomédicas, Facultad de Veterinaria, Universidad Cardenal Herrera-CEU, CEU Universities, Calle Tirant lo Blanc, 7, 46115 Alfara del Patriarca, Spain; laura.lorenzorebenaque@uchceu.es (L.L.-R.); p.catala@cecav.org (P.C.-G.); s.sevilla@cecav.org (S.S.-N.); 2 Chemical Engineering Department, Loughborough University, Loughborough LE11 3TU, UK; D.J.Malik@lboro.ac.uk; 3 Centro de Calidad Avícola y Alimentación Animal de la Comunidad Valenciana (CECAV), 12539 Castellón, Spain

**Keywords:** bacteriophage, *Salmonella*, poultry, Eudragit^®^, microencapsulation

## Abstract

**Simple Summary:**

Bacteriophages are viruses that kill targeted bacteria and could be used as a therapy against multidrug-resistant bacteria in animal production. Gastrointestinal tract conditions throughout the broiler production cycle might compromise the efficacy of bacteriophage oral administration against *Salmonella*. Microencapsulation of phages could protect and prevent the premature release of the bacteriophage, thereby allowing targeted delivery to the colonization site of *Salmonella*, the caecum. This study was designed to assess the optimal timing of the phage intervention over a 42-day production cycle and to compare microencapsulated (delivered in animal feed) and non-encapsulated phages (delivered through the drinking water) delivery along the gastrointestinal tract. Results of this study suggest that microencapsulation of the phages in a Eudragit^®^ L100 pH-responsive formulation allowed targeted delivery of the phage to the chicken caecum. Microencapsulation of phages administered orally through animal feed could be a promising method to control *Salmonella* in the field at any time during the animal rearing period.

**Abstract:**

Bacteriophage therapy is being considered as a promising tool to control *Salmonella* in poultry. Nevertheless, changes in gastrointestinal tract environmental conditions throughout the production cycle could compromise the efficacy of phages administered orally. The main objectives of this study were to assess the optimal timing of the phage administration over a 42-day production cycle and to compare microencapsulated and non-encapsulated phages and the spatial and temporal dynamics of the phage delivery along the gastrointestinal tract. Phage FGS011 was encapsulated in the pH-responsive polymer Eudragit^®^ L100 using the process of spray drying. At different weeks of the chicken rearing period, 15 broilers were divided into three groups. Over a period of 24 h, group 1 received non-encapsulated phages (delivered through drinking water), group 2 received microencapsulated phages (incorporated in animal feed), and group 3 did not receive any phages. Microencapsulation was shown to enable efficient delivery of the bacteriophages to the animal gut and cecum throughout the animal rearing period. During the six weeks of application, the crop displayed the highest phage concentration for both phage delivery methods. The L100 based encapsulation offered significant protection to the phages from the harsh environmental conditions in the PV-Gizzard (not seen with phages administered in drinking water) which may help in the delivery of high phage doses to the cecum. Future *Salmonella* challenge studies are necessary to demonstrate the benefits of microencapsulation of phages using L100 formulation on phage therapy in field studies during the rearing period.

## 1. Introduction

Nontyphoidal *Salmonella* is considered one of the main causes of foodborne outbreaks; it is responsible for around 70 million worldwide cases of human illness and 58 thousand deaths each year [1,2]. In the European Union, despite strict measures carried out by the National *Salmonella* Control Programs (NSCP) in the poultry sector, new cases continue to emerge every year [3,4,5]. When the bacteria come into contact with the birds, *Salmonella* colonizes the gastrointestinal tract (GIT) and spreads to the environment through faeces [6]. For this reason, *Salmonella* colonization is particularly important at three points of the production cycle: During the first week of rearing when the immune system of the animals is still immature [7], the mid-cycle (around 4 weeks old), when *Salmonella* sampling control takes place at farm level [3,8], and at the end of the production cycle (around 6 weeks old), just before the transport of the animals to the slaughterhouse [8]. Thus, the development of effective management strategies, including improved biosecurity measures, vaccination, use of organic acids and prebiotics to improve animal gut health and use of bacteriophages could all help to control the bacteria at the farm level, while maintaining animal health and welfare [9].

In poultry, bacteriophages (BPs) are increasingly being considered as a potentially viable method to enhance animal health and *Salmonella* control through the food chain [10,11]. BPs are viruses whose life cycle is strictly associated with prokaryotic cells [11,12]. Their ubiquitous nature, specificity, prevalence in the biosphere, and low inherent toxicity, make them a safe, natural, and sustainable technology as specific narrow-spectrum antimicrobials [11,12]. A number of previous studies have assessed efficient and cost-effective administration routes and the timing of BP application to improve *Salmonella* control in livestock [13,14]. Phage therapy [13,15,16] is considered safe and especially useful against antibiotic-resistant bacteria [17]. Despite several trials with BPs reporting success in the reduction of *Salmonella* at the field level [9], more research into its effectiveness under commercial conditions is still needed in the poultry sector [18]. Oral administration of BP has previously been shown to successfully treat GIT and systemic infections, however, the effects may be transient and age dependent. In addition, the dosing interval may be a critical factor for the successful implementation of phage therapy [19]. The possibility of BP administration via incorporation in feed or through drinking water would make phage therapy suitable for treatment *en masse*, overcoming a major limiting factor for large-scale poultry applications [20]. Nevertheless, changes in GIT conditions throughout the production cycle could compromise the efficacy of the orally administered BP, leading to variable efficacy outcomes [21,22,23]. In this sense, controlled-release formulation technology has gained interest due to the capability of delivering therapeutics at the target site where they are needed to control the pathogen [24]. Controlled release dosage forms could delay the release of the drug substance in the first stretch of the GIT (crop, proventriculus (PV) and gizzard), reaching the gut and the cecum [23,24]. This way, high doses of BP will reach the target site where *Salmonella* mainly colonizes (the caecum) improving the effectiveness of phage therapy.

The main objectives of this study were to assess the effect of the phage intervention over a six-week production cycle and to compare microencapsulated and non-encapsulated phages and the spatial and temporal dynamics of the BP delivery along the GIT during the chicken rearing period.

## 2. Materials and Methods

The *in vivo* study was carried out in accordance with the recommendations of European Commission (2010/63/CE and 2007/526/CE) and the Spanish legislation (RD 53/2013) [25]. Protocols were designed to comply with the European policy on the “3 Rs” (Replace, Reduce and Refine) in animal experimentation.

### 2.1. BP Origin and encapsulation

BP FGS011 used in this study was isolated by Sevilla-Navarro et al. [26] and characterized by Lorenzo-Rebenaque et al. [23]. BP FGS011 was propagated on *Salmonella* Senftenberg (*S.* Senftenberg), obtained from poultry farms during the NSCP [27] in the Poultry Quality and Animal Nutrition Centre of the Valencia Region (CECAV). During this study, the BP FGS011 was evaluated without encapsulation as free BP (FP) and micro-encapsulated with the polymer Eudragit^®^ L100 (L100) (Evonik Nutrition & Care GmbH, Darmstadt, Germany). Encapsulation was performed according to Malik [28] and Lorenzo-Rebenaque et al. [23]. For this study, the anionic polymer Eudragit^®^ L100 was used for the BP encapsulation. The polymer Eudragit^®^ L100 is insoluble in acid medium, dissolving at pH 6 and greater [29].

Commercially available Eudragit polymer L100 has been specifically designed for enteric delivery applications with the aim of protecting therapeutics from gastric acidity and allowing controlled release of therapeutics utilising a pH-dependent trigger mechanism. L100 is a copolymer of methacrylic acid and methyl methacrylate with different amounts of carboxylic acid residues providing differences in pH dissolution characteristics, the ratio of free carboxyl groups to ester groups is 1:1 [30].

In order to dissolve Eudragit^®^ L100, the pH of the water was changed to alkaline (pH 12) via the addition of 4 M NaOH (Fisher Scientific, Hampshire, UK) to allow polymer dissolution, followed by pH adjustment to pH 7 using 0.1 M HCl prior to addition of trehalose powder (Fisher Scientific, Hampshire, UK), its dissolution, and further addition of bacteriophages to the solution. Typically, 10% (*v*/*v*) high-titre phage (~10^10^ PFU/mL) was added to the solution, yielding phage titres of ~10^9^ PFU/mL in the final formulation. The phage-containing solutions were spray-dried using a commercially available Labplant spray-dryer SD-06 (Labplant, UK Limited), which is a co-current dryer with a pneumatic atomiser and a cylindrical drying chamber of dimensions 215 mm outer diameter and 420 mm height. The diameter of the atomization nozzle used throughout the work was 0.5 mm with the measured feed liquid flow rate at 280 mL·h^−1^ and a drying gas air flow rate of ~20 L·s^−1^. The air inlet temperatures were set at 100 °C resulting in corresponding air outlet temperatures of 60 ± 2 °C respectively.

### 2.2. Experimental design

The study was performed in an experimental poultry house (A) in the Centre for Animal Research and Technology (CITA, IVIA, Segorbe, Spain). A total of 90 1-day-old *Salmonella* free chicks (Ross, males), provided from the same hatchery, were housed to simulate real production conditions. The house was supplied with wood shavings as bedding material, programmable electrical lights, automated electric heating and forced ventilation. The environmental temperature was gradually reduced from 32 °C on arrival day to 19 °C at 39 days post hatch [8]. The birds received drinking water and were fed *ad libitum*. Two different age commercial diets were offered to the animals, a pelleted starter diet from arrival until 21 days post hatch (Camperbroiler iniciación, Alimentación Animal Nanta, Spain) and pelleted grower diet from 21 days post hatch to the slaughter day (Pollos crecimiento G, Alimentación Animal Nanta, Spain).

**Figure 1:**
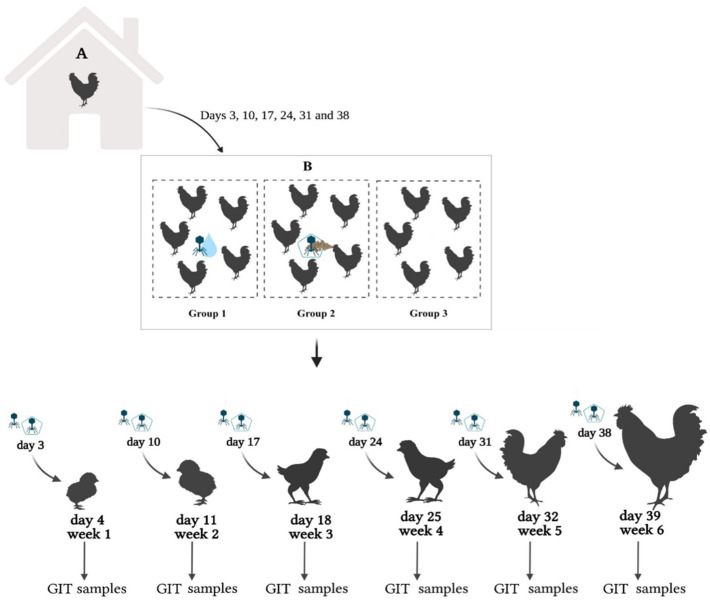
Experimental design of BP application and sampling throughout the entire production cycle. A: control poultry house; B: experimental poultry house; GIT: gastrointestinal tract. Group 1 received the FP via drinking water; group 2 received L100 via feed; group 3 was the control group. Created with BioRender.com.

Once per week, 15 birds were moved to another house (B) and randomly divided into three pens separate by walls in groups of five birds (group 1, 2 and 3). Subsequently, each treatment group was challenged with a single dose of the microencapsulated and non-encapsulated BPs, and after 24 h, animals of each experimental (*n* = 5/group) were slaughtered and sampled. Group 1 received FP at a concentration of 10^8^ PFU/mL via drinking water, group 2 received L100 at a dose of 10^8^ PFU/g via feed, and group 3 did not receive any BP (control group) (Figure 1). Samples were collected from the upper and lower GIT. From the upper GIT, samples from the crop (to assess the percentage of BP entering in the animals) and from the PV-gizzard (to assess acid segments in the BP release) were taken. Concerning the lower GIT, samples from the gut (duodenum, jejunum, ileum, and colon) and the cecum (target segment for *Salmonella* colonization) were taken. Moreover, to assess faecal shedding, at least 10 g of faeces were taken from each experimental group.

### 2.3. Processing of GIT samples

The GIT and faeces samples were analysed according to Lorenzo-Rebenaque et al. [23]. Briefly, samples were weighed and emulsified individually in LB broth supplemented with salts (Luria Bertani, VWR Chemicals, Barcelona, Spain) at 1:10 (*w*/*v*). The samples were centrifuged at 16,000× *g* for 5 min and filtered through 0.45 µm. The BP concentration was measured in each sample using the spot test by the double overlay agar plaque assay method. Thus, ten-fold serial dilutions were performed using sterile dilution buffer (LB), these were spotted onto the surface of bacterial lawns. For this purpose, 200 µL of a log-phase culture of the bacterial suspensions in LB, at an optical density (OD) 600 nm of 0.2 (~10^8^ CFU/mL) was added to 5 mL of molten LB agar (LB with 0.6% (*w*/*v*) agar) tempered set at 45 °C and poured onto previously prepared and dried LB basal agar (with 1.6% (*w*/*v*) agar). Plates were incubated overnight at 37 °C. BP titration was performed per triplicate.

### 2.4. Statistical Analysis

Concentrations (PFU/mL) of BP were converted to Log10 (PFU/mL) [31]. A univariate general linear model was used to access and compare the dynamics of the BP (FP and L100) along the GIT including as fixed-effects factors the gastrointestinal localization (crop, PV-gizzard, gut, and ceca), the application week (weeks 1 to 6), and the BP form (FP and L100), and as a random-effect factor for the different replicates. A *p*-value < 0.05 was considered indicative of a statistically significant difference. Statistical analyses were performed using SPSS 16.0 software package (SPSS Inc., Chicago, IL, USA).

## 3. Results

No adverse clinical signs in animals were observed during the entire experiment. The performance parameters (body weight, feed intake, daily gain, and feed conversion ratio) obtained were in accordance with breeding standards (Ross, 2020). No statistically significant differences were observed between the treatment groups and the control group.

### 3.1. BP Gastrointestinal Dynamics in Chickens According to the Week and Form of BP Application

#### BP Concentration in the First Section of the GIT (Crop and PV-Gizzard)

**Figure 2:**
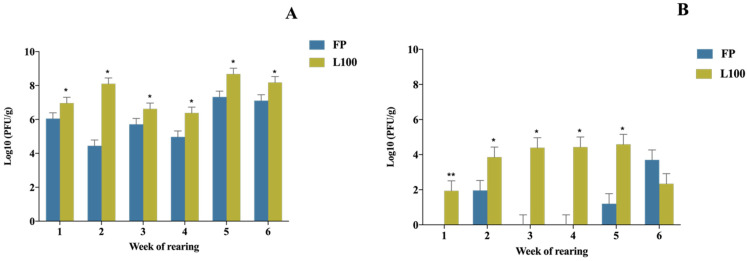
Concentration of BPs recovered following administration depending on the week of application and organ analysed: (**A**) the crop, (**B**) the PV-gizzard. Values are presented as Log10 (PFU/g). Error bars show one standard deviation. The statistically significant differences between groups comparing BP delivery method have been represented as * (*p*-value < 0.05) and ** (*p*-value < 0.001).

Statistically significant differences in phage concentrations were found between the organs (crop and PV-gizzard), the BP delivery method (FP and L100) and the application week (1 to 6) (*p*-value < 0.05). In the crop, statistically significant differences were found between different weeks. The highest phage recovery for both delivery methods was at the end of the production cycle (wk. 5 and wk. 6). Regarding the concentration of BP recovered at each point of sampling, the concentrations of FP obtained were significantly lower than for the microencapsulated BP regardless of the week of application (*p*-value < 0.05) (Figure 2A). In the PV-gizzard, the free/encapsulated BPs counts were lower than those compared to the crop (*p*-value < 0.05). Significantly higher concentrations of phages were recovered from PV-gizzard for the microencapsulated BP L100 compared with free phage treated animals, regardless of the application week (*p*-value < 0.05), except in the sixth week (*p*-value < 0.05) (Figure 2B).

### 3.2. BP Concentration in the Gut and Ceca

**Figure 3:**
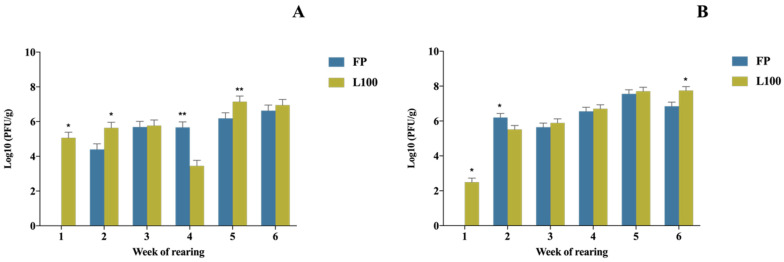
Concentration of the BPs recovered following administration depending on the week of application and organ analysed: (**A**) the gut and (**B**) the cecum. Values are presented as Log10 (PFU/g). The statistically significant differences between groups comparing BP delivery method have been represented as * (*p*-value < 0.05) and ** (*p*-value < 0.001).

Statistically significant phage concentration differences were observed in the gut and ceca (FP and L100) during the treatment period (*p*-value = 0.000). Phages were recovered from animals in the FP treated group throughout the treatment period except for the 1st application week. BP concentrations were observed to increase over time for both delivery methods as the treatment period progressed. Higher counts of BP were measured at the end of the production cycle (Figure 3A,B).

### 3.3. Fecal BP Excretion Profile

**Figure 4:**
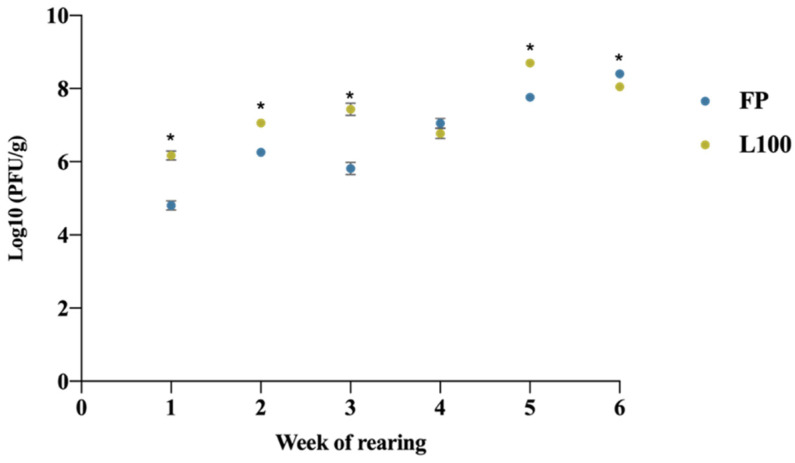
Concentration of the BPs found in faeces depending on the week of application. Values are presented as Log10 (PFU/g). FP: free BP; L100: encapsulated BP with the polymer Eudragit^®^ L100. The statistically significant differences between groups comparing BP delivery method have been represented as * (*p*-value < 0.05).

Higher concentrations of phages were measured in faeces for the encapsulated form (*p*-value < 0.05) when comparing the different BPs delivery methods (Figure 4). The exceptions were for the fourth and last week of the rearing.

## 4. Discussion

BP therapy is considered a promising tool to control *Salmonella* in poultry [10,11]. The easy implementation, host-specificity and cost-effectiveness of phage therapy have resulted in increased interest in BP application at the field level [11,32,33,34]. Previous studies have reported positive results regarding the delivery of microencapsulated *Salmonella* BP in a simulated gastrointestinal model and in 1-day-old chicks [23,28]. The present research is the first study that addresses the spatial and temporal effects of microencapsulated and non-encapsulated BPs delivery at different ages of application during the chicken rearing period. These data suggest that microencapsulated BPs formulated using the polymer Eudragit^®^ L100 have the potential to be used throughout the six-week chicken rearing period.

Results obtained in this study demonstrate the efficient protective effect of L100 delivery through the GIT. The FP administered in drinking water were substantially inactivated in the PV-gizzard conditions however, they remained active and bioavailable in the crop which is a reservoir for infectious *salmonella*. The first week of rearing represents a critical moment for the chicks, as the immune system of the animals is still immature, facilitating the rapid colonization and multiplication of *Salmonella,* thereby affecting the entire production cycle [7,35,36,37]. Protection in young animals in which the immune system and GIT microbiota are not fully mature could ensure a *Salmonella*-free flock at the field level [38].

Results of our study during the first week of life are in agreement with previous reports in which no antimicrobial effects were observed after the application of oral therapy [16,39,40]. Nabil et al. [16] reported the need for several BP doses to obtain *Salmonella* reduction, however, the lack of BP effectiveness during the first week of rearing could be due to the low PV-Gizzard pH and short retention times in the chick’s intestinal tract during the first 7 days of its life, that prevents the BP from reaching the *Salmonella* colonization site [41].

The application of BPs from the second week onwards showed that regardless of the delivery method (FP and L100), some of the administered BP dose was able to reach the gut and cecum. The effect of the encapsulation allowed L100 to overcome the adverse environmental conditions of the PV-gizzard allowing higher doses of viable phages to reach the caecum. A small amount of the FP could also pass through the GIT perhaps protected by the buffering effect of the feed [21,42,43,44].

The highest BP concentration was mainly obtained in the crop regardless of the delivery method applied however, free phages applied in drinking water would bioavailable in the crop unlike encapsulated phages. These results are in line with those previously reported, where a difference of up to three log10 was found between the crop and the gut-cecum concentrations [44]. The prolonged retention of high doses of BP administered in drinking water present in the crop may provide protection to any new orally ingested pathogen, such as *Salmonella* [22,44]. The crop is considered, together with the cecum to be the major site of *Salmonella* colonization in the chicken [45]. The long BP residence times in the crop would allow phage-host interaction with potential phage amplification [44]. Moreover, it was shown that for both FP and L100 delivery methods, high concentrations of BP were present in the cecum. A hypothesis that could explain the high concentration at the end of the GIT may be the ability of BP predation on non-target species [46]. The previous host range characterization of the BP FGS011 demonstrated its capability to lyse *Citrobacter* [23], known to be associated with poultry microbiota and *Salmonella* epidemiology [47,48]. This lysis against *Citrobacter* enhances the possibility of BP co-evolution in the gut that may lead to increases in BP concentration [46]. Thus, the possibility to control *Salmonella* in the intestinal tract of chickens before slaughter by the application of BP may prevent carcass contamination during the slaughtering process and reduce the risk of *Salmonella* transmission via contaminated chicken meat to consumers [49].

Excretion of the phage in animal faeces, along with the presence of the phage in feed and/or water may result in the presence of the phages in the house environment, facilitating re-infection of animals with the phage, and the protection of animals from future bacterial challenges [33,44,50].

The results of this study highlight the importance of BPs survival dynamics following their administration through the GIT in drinking water and animal feed. The oral route was chosen for ease of administration and delivery of the phage with non-encapsulated phages given via drinking water and encapsulated phages in the animal feed. BPs have been administered via feed and drinking water previously. This is a feasible low-cost delivery method for large-scale application in poultry farms [51,52,53]. Lim et al. [51] and Vaz et al. [53] applied the BPs in the feed and water, respectively to reduce *Salmonella* colonization in broilers. The administration route (feed or water) as well as the delivery method (encapsulated and non-encapsulated) was shown to affect the survival of the phages in the PV-Gizzard and could impact the efficacy of phage therapy. Other GIT environment factors, such as intestinal volume, local pH variation, viscosity and presence of commensal microbiota, could affect the BP concentration at the target site, resulting in differences in BP-host interactions [53]. Combination of BPs with dietary supplementation with probiotics have previously been reported [54,55]. A synergistic effect was shown against *Salmonella* infections in broilers [54] and the potential to improve the performance in piglets was noted [55]. This possibility of a combination of phages with other antibiotic alternatives employed in poultry production could be of significant future interest to achieve a higher degree of effectiveness against the bacteria.

## 5. Conclusions

The conclusions of this study highlight that BP encapsulation with the polymer Eudragit^®^ L100, especially when administered at the beginning and at the end of the cycle, could ensure targeted delivery of high titres of phages to the caecum affording encapsulated phages protection from the harsh environmental conditions found in the PV-Gizzard. Moreover, the fact that encapsulated BPs were found in the crop and caecum, known sites of high *Salmonella* colonization, makes encapsulation of phages a promising tool to control the bacteria at the field level. On the other hand, the easy dissemination of the BP through faeces may also facilitate the control of the bacterium in the farm environment. However, further *Salmonella* challenge studies are necessary to evaluate the beneficial effects of encapsulation of phages using L100 formulation added to feed perhaps combined with phages added to drinking water to control the bacteria in the field during the rearing period.

## Data Availability

Not applicable.
